# Supporting Student’s Mental Health: A Cross-Sectional Survey for School Nurses

**DOI:** 10.3390/children8020129

**Published:** 2021-02-10

**Authors:** Pihla Markkanen, Minna Anttila, Maritta Välimäki

**Affiliations:** 1 Department of Nursing Science, University of Turku, 20014 Turku, Finland; pihmar@utu.fi (P.M.); minant@utu.fi (M.A.); 2 Xiangya School of Nursing, Central South University, Changsha 410083, China

**Keywords:** school nurse, public health nurses, school health services, mental health, student

## Abstract

Children’s and adolescents’ health problems are often related to mental health, and their wellbeing should be supported in schools. This study describes school nurses’ role and how equipped they are in recognizing students’ mental health needs and in supporting students’ mental health. Moreover, we explored the methods used and the barriers that exist for supporting students’ mental health. A national survey for members of the Finnish Public Health Association working as school nurses was conducted (*n* = 136/648, 21%). The survey questionnaire was analyzed using descriptive statistics and qualitative data using manifest content analysis. Participants (*n* = 127/133, 96%) agreed that they had an important role in ensuring that students’ mental health needs are met on time. Around one-third reported training needs for mental health interventions (*n* = 42/115, 36%), and a similar proportion (*n* = 42/136, 31%) indicated lacking adequate knowledge and skills for supporting mental health among culturally diverse students. Identified barriers for students getting help were a lack of options for sending students to mental health services (*n* = 92/134, 69%) and a lack of adequate training (*n* = 81/134, 68%). School nurses are key in providing early mental health support to students. Therefore, the availability of intervention education and training on assessing and supporting students’ mental health is needed and should be improved.

## 1. Introduction

Mental health among youth is a serious global concern [1]. Half of all mental health disorders commencing in the mid-teens [2] and a global prevalence of 10−20% was reported for disabling mental illness [1,3,4]. Depression and anxiety are among the most common health concerns for youth [5] and economically burden society [6]. A Finnish study [3] showed that the use of specialized psychiatric and neurodevelopmental services among youth aged 12–18 has increased between 1987 (9.8%) and 1997 (14.9%) birth cohorts. The most significant increase was seen in female diagnoses (depression and anxiety), while the most common diagnosis for males was emotional and social interaction disorders and attention deficit hyperactivity disorder [3].

School health services vary between countries. An overview of 102 countries that included 47 high-income, 26 upper-middle income, and 29 lower-middle and low-income countries reported that in 58% (*n* = 59) of countries, school health personnel worked exclusively in school healthcare, while in the remaining countries, healthcare personnel made visits to schools or invited students to primary healthcare clinics. Mental healthcare was offered in 48 countries, less common in lower-middle and low-income countries [7].

In Finland, where inequality in the level of wellbeing is second lowest in the world [8], school concerns based on health examinations are mostly related to students’ psychosocial development and family stressors [9]. According to studies conducted in the US, Sweden, the UK, and Norway, school nurses can provide brief interventions or refer students to mental health services [10,11,12,13]. A study in the US showed that 75% of school nurses (*n* = 186) offered emergency services for child abuse, neglect, depression, or violence at school, and over 40% for suicidal students [14].

At the same time, school nurses may feel unprepared in supporting students’ mental health. Clausson et al. (2015) found that Swedish school nurses were uncertain in documenting mental health issues out of concern that it may stigmatize students [15]. School nurses may also be insecure in their ability to recognize depression and in supporting cases of self-harm behavior [13,14,15,16]. School nurses in the US were unsure in addressing high-risk situations, such as students with homicidal thoughts. They reported being more confident in addressing attention problems or intervening in child abuse [17].

Norwegian school nurses were reported to desire training in motivational interviews that support self-esteem [13]. An intervention study in the UK showed that school nurses benefitted from short courses on depression; based on the three-month follow-up, school nurses’ knowledge on depression improved (intervention *n* = 61, control *n* = 54, *p* = 0.002) and their confidence increased (*p* = 0.004) [18]. However, Haddad et al. (2010) showed that nearly half of school nurses had not had continuing education in mental health [12]. A survey in the US also showed that although 76.3% of the school nurses (*n* = 93) reported that anxiety was the most prevalent mental health issue among students, they did not use any evidence-based instruments or interventions to assess or manage students’ anxiety [19]. According to a pilot randomized controlled trial [20], a brief intervention for students with anxiety (*n* = 54), provided by school nurses (*n* = 30), had a positive impact and reduced anxiety and related symptoms.

In Finland, schools and student healthcare services are equal in quality and availability across the country [8]. Student health services are free of charge at the primary, secondary, and upper secondary school levels and inexpensive at the university level. School nurses are expected to observe both the physical and mental wellbeing of students [8]. The Ministry of Social Affairs (2004) recommends at most 600 students per one full-time school nurse [21]. Youth (87%, *n* = 7186) have evaluated access to a school nurse to be easy [22].

According to earlier international studies school nurses can deliver mental health interventions [10,13,16], but they are not always recognized as part of the school mental health workers. In Finland, school nurses meet all the students systematically as they run regular health examinations, and they are available for students during the school days. There appear to be no studies in a Finnish context about how school nurses are equipped in recognizing students’ mental health needs and supporting students with mental health problems. We aim to describe in detail the role of school nurses in supporting students’ mental health at school, what mental health problems school nurses have identified and how they have prepared themselves in supporting students’ mental health, interventions used, and barriers for supporting students’ mental health. These results can provide information in countries where school nurses have a similar role as in a Finnish school setting. Results can also be utilized in middle- and low-income countries, especially when they reform school health services, tailor services to their own specific needs, and improve the role of the school nurse as a potential mental health worker at school. School nurses have an important role as they are the most widely reported professionals working in school health services, and thus they are an important part of health systems [23]. Baltag et al. (2015) pointed out in their overview of 102 countries that school health service is one of the only services that reaches the majority of adolescents on an almost daily basis and is particularly well placed to reach adolescents with preventive interventions [6].

## 2. Materials and Methods

### 2.1. Setting

In Finland, the school and student welfare services are organized by the municipality [8]. Compulsory schooling consists of one year of pre-primary education and nine years of basic education starting the year the child turns seven. Comprehensive school comprises primary school (grades 1–6) and junior secondary school (grades 7–9). After comprehensive school, most youth continue in general/vocational upper secondary education [24], in 2019, 94% did so [25]. The higher education system comprises universities and universities of applied sciences [24].

### 2.2. Population and Sample

The study population was identified from the member register of the Finnish Public Health Nursing Association, founded in 1938. It is a voluntary-based professional association for public health nurses and audiologists. In 2018, the association had 7500 members, *n* = 4800 (64%) were public health nurses, while 16% (*n* = 766) worked at schools [26].

### 2.3. Participant

School nurses in Finland are qualified public health nurses with a bachelor’s degree from a university of applied sciences (four-year studies) [27]. Inclusion criteria for the participants were (1) being a member of the Association, (2) working as a school nurse, (3) and having an email address registered with the Association. Members of the Association who did not work as a school nurse at the time of data collection were excluded.

### 2.4. Instrument

Data were collected with the Mental Health Needs and Practices in Schools Survey, an instrument developed and validated in the US [28,29]. After a search of the literature, the instrument was selected as it covers roles and practices among school nurses related to students’ mental health at school. It originally included 42 questions, but in this study, 29 questions relevant in a Finnish context were used. This decision was approved by the developer of the instrument.

After permission to use the instrument was granted, all items were translated from their original English to Finnish, then blindly back-translated from Finnish to English by a professional translator [30]. After that, the agreement between the original and back-translated versions was considered, and the translation process was continued until the equivalency in meanings was ensured. The instrument was adapted for the work of school nurses in Finland by orientating it toward the Finnish welfare system in terms of culture and education [30,31]. For example, a question asking what kind of data is maintained at school was left out because school nurses have access to students’ medical records in Finland. Some background questions, e.g., professionality or school name, were left out. We included the questions asking, from the school nurses’ viewpoint, if ‘Teachers should be involved in addressing the following mental health needs of the students’ in the survey, because teachers work closely with students every day. They may also do the first initiative to send students to the school health services and collaborate with school nurses [32]. When the back-translated version was considered appropriately equivalent to the original version by the authors, the developer confirmed the translated instrument and its cultural adaptation.

The final version of the instrument was pretested with school nurses (*n* = 4, non-members of the Association) working in primary and secondary schools to ensure face-validity [33] (pp. 377–378). They were asked to comment on the understandability and suitability of the items [34]. According to their feedback, the instrument was easy to understand, and the questions were relevant to their work. The items and the way they were asked are described below and in Table 1, Table 2, Table 3 and Table 4.

### 2.5. Content of the Instrument

#### 2.5.1. Background Characteristics

Respondents were asked their background information. They were asked to indicate which persons currently work in their school and the time devoted to one school.

#### 2.5.2. The Role of School Nurses and Schools in Supporting the Mental Health of Students

Respondents were asked to use a 5-point Likert Scale (1 = strongly disagree to 5 = strongly agree) to indicate the extent to which 13 items (e.g., having a role in ensuring that the mental health needs of students are met on time) characterized their role or attitude regarding mental health interventions and practices.

Again, using the same 5-point Likert-Scale, respondents rated eight statements (e.g., screening of mental health problems) related to the extent they felt that teachers should be involved in addressing students’ mental health needs. The internal consistency of the subscale has been adequate in a previous study (Cronbach’s alpha 0.78) [28], and in our study it was 0.71.

#### 2.5.3. Identifying Mental Health Problems among Students

Respondents were asked to indicate whether they had worked with a student in the past year who was affected by any of the 15 different items (e.g., depression) by answering “yes” or “no”. They were further asked to name most concerning mental health issues in their school. In addition, they were asked to indicate the number of students and families they had referred to mental health services in the past school year. They were also asked to indicate the top three challenges in their work.

#### 2.5.4. Preparedness to Support Students’ Mental Health

With three questions, respondents were asked to assess their level of knowledge and skills required to meet the mental health needs of students. Moreover, with a 5-point Likert Scale (1 = strongly disagree to 5 = strongly agree), they assessed the adequacy of their cultural knowledge and communication/interpersonal skills for meeting the mental health needs of culturally diverse students.

Further, the respondents were asked to rate their own education or training in using behavioral interventions and the amount of experience in using it with a 4-point Likert-Scale (1 = none to 4 = substantial). The respondents were then asked to report in eight questions (e.g., education during their nursing studies) what kind of training they had received in behavioral interventions and in what format the training was received. In addition, they were asked, with an open-ended question, the top three areas in which they felt they needed additional training.

#### 2.5.5. Mental Health Services and Interventions Used in School

Respondents were asked about current services provided in their school, with 15 items (e.g., individual counselling) that they were to answer with “yes,” “no”, or “not sure”. Additionally, school nurses were asked open-ended questions about the mental health support programs and services at their schools that would support student’s mental health.

#### 2.5.6. Barriers in Mental Health Support at Schools

Respondents were asked about the barriers in providing students’ mental health services in schools, rating 12 items (e.g., difficulties in identifying students’ mental health needs) using a 5-point Likert scale (1 = strongly disagree to 5 = strongly agree). The internal consistency of the subscale has been adequate in a previous study (Cronbach’s alpha 0.82) [28], in our study it was good: 0.89.

With the same 5-point Likert-Scale, respondents were asked about why students with mental health needs might not get the help they need. This section included 10 items. The internal consistency of the subscale has been adequate in a previous study (Cronbach’s alpha 0.86) [28], in our study it was also adequate: 0.86.

### 2.6. Procedure

A cross-sectional study design was used in this descriptive study. The invitation email was sent to Association members working as school nurses (*n* = 648). The online survey method was chosen because of its quickness, inexpensiveness [35], and participants could answer at their convenience [36].

All 648 school nurses received an email invitation, which included information about the study and the researchers’ contact information for further questions. Willing participants were instructed to use the link in the invitation, which guided them to the electronic survey. On the first page of the survey, there was more information about the study aims, voluntary participation, confidentiality, and anonymity. Participants were informed about their rights to refuse or withdraw participation at any stage [37]. Participants were then invited to give their informed consent by answering “yes” or “no.” If they answered “yes,” participants were able to answer the survey electronically.

The recruitment process was conducted between 3 April and 1 June, 2018. Five reminder emails were sent to all possible participants [38] and two reminders were sent by the Association through Facebook. Out of 648 possible participants, 137 provided their informed consent. One participant only answered the background questions and was excluded from the analysis. This left us with 136 completed forms (response rate of 21%).

### 2.7. Data Analysis

The data were first analyzed using descriptive statistics (frequencies, percentages, means, standard deviations) by following the analysis strategy of the original publications [28,29]. The quantitative data were analyzed using SPSS version 25 [39]. Participants’ answers to the open-ended questions were coded and categorized using manifest content analysis [40]. First, the answers were analyzed by coding the data manually and then creating categories. For example, the participants were asked to name most concerning mental health issues in their school, to which most of them answered with a short list. These items were coded, for example: fear of social situations was coded deductively as social anxiety disorder and further added into the category of anxiety. Finally, the items in the categories were interpreted into numerical data and counted [33] (pp. 554–555) and [40].

### 2.8. Ethical Issues

The study proposal was evaluated by the Ethics Committee of the University of Turku (5/2018). Approval of the study was obtained from the Association (7 March 2018). Neither the identities nor email addresses of the potential participants were made known to researchers because the invitations were sent by the Association. Background questions were formulated in such a way that the participants’ anonymity was secured and names of schools or municipalities were not asked.

## 3. Results

### 3.1. Participants

The ages of the participants (*n* = 136) ranged from 24 to 64 years (Mean 47.39, SD 10.49). Almost all (*n* = 134, 99%) were females. The number of years since qualification as public health nurses ranged from 0 to 42 years (Mean 18.5, SD 10.64). Most of the participants’ (*n* = 118, 91%) highest level of education was a bachelor’s degree, and less than one-tenth (*n* = 12, 9%) had a master’s degree. Working experience in school nursing ranged from 0 to 37 years (Mean 13.48, SD 8.53). Participants were from throughout Finland, one-third of them (*n* = 46, 34%) were from West Finland or from the Helsinki-Uusimaa region (*n* = 42, 31%), and rest of the participants were from North or East (*n* = 32, 24%) or South Finland (*n* = 16, 12%). Nearly half of participants, *n* = 54 (40%), worked in a municipality with more than 80,000 inhabitants, *n* = 39 (29%) worked in a municipality with 30,000–80,000 inhabitants, *n* = 28 (21%) worked in a municipality with 8000–29,999 inhabitants, *n* = 11 (8%) in a municipality with 4000–7999 inhabitants, and *n* = 4 (3%) in a municipality with less than 4000 inhabitants.

Participants worked at all school levels, some of them at different school levels during the week. Over half of them (*n* = 77, 57%) were working at primary school, *n* = 56 (41%) worked at junior secondary school, *n* = 35 (26%) worked at upper secondary school (vocational education), *n* = 30 (22%) worked at upper secondary school (general education), and *n* = 19 (14%) worked at student healthcare in a university of applied sciences or at a university.

Sixty-five percent of schools (*n* = 107) had a counselor, and in less than one-fifth (*n* = 14, 14%) they worked part-time. About one-third (*n* = 39, 29%) had a full-time psychologist, and in 46% of schools (*n* = 63) they worked part-time. One-third of schools (*n* = 49, 36%) had a full-time social worker, and in half (*n* = 70) they worked part-time. Most schools (*n* = 96, 71%) had a full-time school nurse, and 29% (*n* = 40) had a part-time nurse. In 62% of schools (*n* = 84), there was at least one full-time teaching assistant, and 6% of schools (*n* = 8) had at least one part-time assistant. Five percent of school nurses (*n* = 6) reported that there was a full-time mental health professional, and 12% (*n* = 14) reported a part-time mental health professional in their school.

### 3.2. The Role of School Nurses and Schools in Supporting Students’ Mental Health

Most of the participants indicated that schools should be involved in addressing the mental health of students: 45% (*n* = 61) strongly agreed and 51% (*n* = 61) agreed. School nurses reported their attitudes and role regarding mental health interventions and practices (Table 1).

### 3.3. Identified Mental Health Problems among Students

The school nurses reported if they had worked with students with specific mental health concerns within the last year. The most common issues reported were concentration problems (*n* = 135, 99%), depression (*n* = 134, 99%), anxiety (*n* = 131, 96%) and family stressors (*n* = 129, 95%) (Table 2).

The mental health problems in school that participants found the most concerning were (1) depression or suicidal ideation and behavior (*n* = 132, 98%), (2) anxiety including panic disorder (*n* = 106, 79%), and (3) behavioral problems such as aggressive, disruptive, or impulsive behavior (*n* = 62, 46%).

Within the past school year, 122 (90%) participants had referred students and 92 (68%) had referred families to mental health services. The three challenges that participants (*n* = 131) faced most often were (1) a lack of time and feeling pressure (*n* = 72, 55%), (2) difficulties in referring students to mental health services (*n* = 61, 47%), and (3) challenges related to students’ families (*n* = 35, 27%).

### 3.4. Preparedness to Support Students’ Mental Health

Forty-seven percent (*n* = 63) of the participants agreed and 10% (*n* = 13) strongly agreed they had an adequate level of knowledge, while 41% (*n* = 55) agreed and 13% (*n* = 17) strongly agreed that they had the skills required in supporting students’ mental health. Altogether, 24% (*n* = 32) disagreed and 7% (*n* = 10) strongly disagreed that they had an adequate level of cultural knowledge and interpersonal skills for supporting mental health among culturally diverse students (Table 3).

The school nurses rated the extent to which they felt teachers should be involved in addressing students’ mental health (Table 3).

The respondents reported their education or training in using behavioral interventions to be moderate (*n* = 67, 50%) or minimal (*n* = 50, 37%), and their experience in using behavioral interventions was moderate (*n* = 53, 39%) or minimal (*n* = 54, 40%). They indicated all the types and formats of education they had had. Most common were independent study (*n* = 59, 44%), staff development (*n* = 50, 38%), graduate course work (*n* = 26, 20%), and education during their nursing studies (*n* = 25, 19%). Altogether, 42 (31%) reported not having any education in behavioral interventions. The respondents who provided details of areas where more training was needed (*n* = 115) most commonly reported that skills and tools/interventions for supporting and/or assessing students’ mental health (*n* = 42, 37%) were needed. The second most common area was cultural knowledge, for example, regarding the mental health needs of refugees (*n* = 31, 27%). Thirdly, they desired more training in mental health in general (*n* = 27, 23%).

### 3.5. Mental Health Services and Interventions Used in School

Almost all (*n* = 131, 96%) reported that wellbeing was promoted in their schools. Individual counselling was offered in 93% of schools (*n* = 126). In-home mental health services offered by school personnel were used the least (*n* = 20, 15%) (Table 2).

School nurses (*n* = 75) reported that there are interventions and programs used in their schools that supported students’ mental health. The program named most often was an antibullying program (*n* = 19, 25%). The most commonly mentioned methods were theme days (*n* = 22, 33%), for example, mental health day or positivity week. School nurses (*n* = 87) reported the methods/interventions they had used in the past year to support students’ mental health: collaboration with student welfare services (*n* = 59, 68%) and use of internet-based materials (*n* = 22, 25%) when working with students, for example, materials of the Finnish Association of Mental Health or mentalhub.fi.

### 3.6. Barriers to Supporting Students’ Mental Health

Participants reported reasons why they felt that students might not get the mental health help they need. A lack of prevention programs for students with internalizing behavior problems or extrovert behavior problems were the most agreed reasons. The most agreed (*n* = 61, 46%) or strongly agreed (*n* = 31, 23%) barrier was the lack of options for sending students to mental health services (Table 4).

## 4. Discussion

Our study shows that school nurses have an important role in working with students with mental health problems and they seemed to be aware of the mental health problems in their schools. Despite more than half of the participants reporting that they had adequate knowledge (55%) and skills (53%) for supporting students’ mental health, a similar proportion had concerns about whether the training was adequate when indicating reasons for students not getting help (48%) and barriers in mental health services in schools (60%). These results may be contradictory for various reasons. For example, the participants may have assumed that a lack of adequate training referred to all personnel working in schools rather than just school nurses. Moreover, the questions about having adequate knowledge and skills for supporting students’ mental health were asked in the beginning of the survey. At this point, most of the participants indicated having adequate knowledge and training. Later in the survey they were asked more detailed questions related to students’ mental health. Answering the survey may have highlighted the need for training for school nurses in mental health interventions. The results of this study are based on self-reported perceptions; in the future, it would be useful to study the capacity of school nurses to support students’ mental health using more diverse methods. The need for knowledge and training related to students’ mental health has also been recognized in earlier studies [12,13,14,16]. Earlier studies have pointed out that school nurses may feel unconfident in recognizing depression or supporting students with self-harm behavior [12,16]. In our data, the most commonly mentioned knowledge gap was in how to use instruments and interventions in supporting and/or assessing students’ mental health.

About one-third felt that they did not have adequate cultural knowledge and skills required for supporting the mental health of culturally diverse students. Cultural minorities in Finland are Swedish-speaking Finns, the Roma, Ingerian Finns, Karelian speakers in Finland, Jews, and Tatars. The Saami are recognized as indigenous people. According to the School Health Promotion Study, which is delivered nationally in Finland in every two years, students with immigrant background had more difficulties in receiving student welfare services compared to non-immigrant students [41]. Supporting students with an immigrant background is of topical importance, since the number of people seeking refugee status in Europe has been at a record high since 2015 [42]; there were 11,000 refugee minors in Finland and many of them came alone to the country. According to a study in Finland, the access and content of student healthcare for asylum seekers varies among municipalities [43]. Moreover, the prevalence of mental health problems among asylum-seekers or refugee minors in Europe is high [42,44].

According to the results, school nurses indicated that teachers should carry out behavior-related interventions (96%) and teach social and emotional skills (95%) to students, but screening students’ mental health status was not considered to be the teachers’ responsibility (40%). The differences in nurses’ and teachers’ roles are generally clear. Still, some teachers may assume that their role is to support students’ learning rather than all areas of their personal growth, which may cause debates between nurses and teachers as school nurses are expected to observe both the physical and mental wellbeing of students [8,45]. Participants also reported that the main challenges in school nurses’ work were a lack of time and feeling work pressure, and difficulties in referring students to mental health services. This was also seen as a barrier to getting students the needed help. The workload of school nurses should be addressed so that they could be available for students and also have opportunities to participate in vocational education. In earlier studies, school nurses have highlighted the importance of being available for students [13,16]. In Finland, school nurses are familiar with most of the students since they run regular health examinations and meet all students in their school. However, in smaller schools they are only present part of the week. Still, Kivimäki et al. (2019) found that youth generally reported that accessing their school nurse was easy, even though this varied between the schools [46]. The open-door policy of school nurses is important for students, as they are often the only school health professionals students can go to without a scheduled appointment.

School nurses reported that collaboration with student welfare services and using internet-based materials are methods they use in supporting students’ mental health. They seek internet-based sources, e.g., from mentalhub.fi, that provide information, materials, and knowledge [47]. School nurses provide important support to students with mental health problems in their everyday work, but the lack of education about interventions and a lack of time and resources inhibit the utilization of their full potential. Since school nurses can be critical in providing early mental health support, pre-service training and vocational education about interventions for assessing and supporting students’ mental health should be available. Internationally, as mental health services are less common in middle- and low-income countries and they are often provided in a context of projects rather than routine service provision [7], it is important that they will be given sufficient consideration in routine service provision. Skundberg-Kletthagen and Moen (2017) have also pointed out that school nurses need to be considered the “best-placed” professionals to provide early mental health help to adolescents [13]. To become such a resource, they need confidence in knowledge and skills concerning mental health problems and support and supervision to be able to help and strengthen adolescents’ mental health.

### Limitations

Results are based solely on school nurses’ self-reported answers describing their own perceptions, rather than from the perspective of students. The sample size is limited since the response rate was only 21%. There was selection bias present in the sample [33] (p. 383). Those school nurses who responded to the survey may have been more likely to address mental health issues or have more experience or interest in treating them. It is also possible that school nurses who did not respond may have had views that were not adequately represented in this sample. For example, some of those school nurses who did not participate may have considered that mental health support of students is not a priority for them. Moreover, results are not generalizable to other countries even though the participants represented school nurses with a wide range of working experience time, throughout Finland, from all school levels. The strengths are that the 136 participating school nurses were responsible for approximately 80,000 students and had firsthand knowledge about their wellbeing. We must also state that the school nurses were from a wide range of school levels (primary to university), and primary school nurses have different roles in adolescents’ mental health relative to school nurses working at the university, making it difficult to make conclusions.

## 5. Conclusions

This national survey for school nurses showed their important role in supporting students with mental health problems. They have an adequate level of knowledge and skills in supporting mental health, but they need more knowledge about interventions, assessment methods, and ways to support culturally diverse students. At present, the complete capacity of school nurses in supporting students’ mental health may not be utilized because of the lack of available interventions and resources. In the future, more research about the effectiveness of mental health interventions in school and student healthcare is needed. It is also important that up-to-date education and training are available for school nurses.

## Figures and Tables

**Table 1:** School nurses’ attitudes and role regarding mental health interventions and practices.

	*n*	Strongly Disagree *n* (%)	Disagree *n* (%)	Neutral *n* (%)	Agree *n* (%)	Strongly Agree *n* (%)
Having a role in ensuring that students’ mental health needs are met on time	133	0	1 (1)	5 (4)	65 (49)	62 (47)
Willing to try new types of interventions	133	0	4 (3)	19 (14)	70 (53)	40 (30)
Relying on own judgement when adopting a new practice	133	0	3 (2)	48 (36)	74 (56)	8 (6)
Adopting practices that have been proven to be effective	133	0	2 (1)	34 (26)	71 (53)	26 (20)
Being aware of the issues that need to be considered when selecting practice	131	1 (1)	3 (2)	47 (36)	70 (54)	10 (8)
Research-based interventions lack feasibility at schools	131	4 (3)	19 (15)	80 (61)	26 (20)	2 (2)
Experience is more important than the use of interventions	132	4 (3)	29 (22)	65 (49)	32 (24)	2 (2)
Would not use manualized intervention	131	10 (8)	56 (43)	60 (46)	4 (3)	1 (1)
Would try a new type of intervention	131	0	2 (2)	28 (21)	86 (66)	15 (12)
Confident that the interventions/practices used have the desired impact	132	0	7 (5)	76 (58)	46 (35)	3 (2)
Having the appropriate knowledge in selecting and implementing the interventions	132	12 (9)	43 (33)	42 (32)	32 (24)	3 (2)
Having the resources to select and implement the interventions	133	20 (15)	65 (49)	30 (23)	18 (14)	0
Gathering data of benefits of the interventions	132	12 (9)	36 (27)	42 (32)	42 (32)	0

**Table 2:** Reported student mental health concerns within past year and mental health services and interventions used in schools.

**Concern**	*n*	***n*** **(%)**
Concentration problems	136	135 (99)
Depression	135	134 (99)
Anxiety issues	135	131 (96)
Family Stressors	136	129 (95)
Friendship issues	136	122 (90)
Hyperactivity	136	117 (86)
Victim of bullying	135	115 (85)
Bullying	136	111 (82)
Disturbing/impulsive behavior	134	103 (76)
Deviant behavior	136	93 (68)
Problems related to immigration or cultural adaptation	132	84 (64)
Considering dropping out of school	132	81 (62)
Aggressive behavior	134	78 (58)
**Mental health services and interventions provided in schools**	***n***	***n*** **(%)**
Promoting health and wellbeing	134	131 (98)
Individual counselling	135	126 (93)
Service guidance	135	120 (89)
Referral to specialized programs/services	135	97 (72)
Crisis intervention	136	88 (65)
Medication management	133	83 (62)
Assessment of emotional or behavioral problems	136	80 (59)
Group counselling	134	77 (57)
Guidance for parents	135	76 (56)
Consultation in behavior management	134	71 (53)
Early intervention and preventive programs	133	66 (50)
Supporting positive behavior at school	135	66 (49)
Support services for families	134	44 (33)
Disturbing behavior analysis and intervention planning	134	40 (30)
Mental health services offered in the homes	135	20 (15)

**Table 3:** School nurses’ knowledge and skills for supporting student mental health and their perceptions of teachers’ involvement in addressing mental health needs of the students.

	*n*	Strongly Disagree *n* (%)	Disagree *n* (%)	Neutral *n* (%)	Agree *n* (%)	Strongly Agree *n* (%)
**Knowledge and skills in supporting student’s mental health**						
Having the level of knowledge required	135	3 (2)	23 (17)	33 (24)	63 (47)	13 (10)
Having the level of skills required	135	3 (2)	27 (20)	33 (24)	55 (41)	17 (13)
Having the adequate cultural knowledge and interpersonal skills	136	10 (7)	32 (24)	53 (39)	39 (29)	2 (1)
**Teachers should be involved in addressing the following mental health needs of the students**						
Screening of mental health problems	136	10 (7)	44 (32)	39 (29)	34 (25)	9 (7)
Referring to school-based services	136	0	2 (1)	10 (7)	58 (43)	66 (49)
Referring to municipality-based services	135	2 (1)	17 (13)	33 (24)	62 (46)	21 (16)
Carrying out behavior-related interventions in the classroom	134	0	0	6 (4)	34 (25)	94 (70)
Teaching social and emotional skills	136	0	2 (1)	5 (4)	42 (31)	87 (64)
Behavioral assessment	135	0	0	7 (5)	51 (38)	77 (57)
Evaluating student progress	136	0	0	6 (4)	45 (33)	85 (63)
Recognizing parent-/family-based problems	136	6 (4)	16 (12)	37 (27)	57 (42)	20 (15)

**Table 4:** Reasons for students not getting help and barriers for mental health services in schools.

Students’ Needs Are not Met Because of the Lack of:	*n*	Strongly Disagree *n* (%)	Disagree *n* (%)	Neutral *n* (%)	Agree *n* (%)	Strongly Agree *n* (%)
Adequate support for parents	134	4 (3)	23 (17)	39 (29)	48 (36)	20 (15)
Prevention programs for students with extrovert behavioral problems	134	3 (2)	18 (13)	42 (31)	59 (44)	12 (9)
Prevention programs for students internalizing behavior problems	134	2 (1)	29 (22)	22 (16)	58 (43)	23 (17)
Anti-bullying programs	134	17 (13)	45 (34)	41 (31)	27 (20)	4 (3)
Early intervention programs	134	12 (9)	39 (29)	39 (29)	31 (23)	13 (10)
Early screening and pre-referral programs	134	10 (7)	53 (40)	24 (18)	39 (29)	8 (6)
Staff training	134	5 (4)	31 (23)	34 (25)	52 (39)	12 (9)
Adequate crisis planning and support	134	4 (3)	38 (28)	41 (31)	42 (31)	9 (7)
Implementation of existing programs	133	0	22 (17)	62 (47)	38 (29)	11 (8)
Observation of students with mental health needs	132	5 (4)	37 (28)	37 (28)	43 (33)	10 (8)
**Existing barriers to providing mental health services at my school(s):**						
Difficulties in identifying students’ mental health needs	134	17 (13)	55 (41)	25 (19)	34 (25)	3 (2)
Lack of mental health professionals	134	9 (7)	16 (12)	11 (8)	50 (37)	48 (36)
Lack of adequate training	134	7 (5)	24 (18)	22 (17)	60 (45)	21 (16)
Difficulties in getting parents to collaborate	134	10 (7)	34 (25)	47 (35)	37 (28)	6 (4)
Stigma associated with mental health services	134	9 (7)	30 (22)	35 (26)	50 (37)	10 (7)
Language and cultural barriers	133	6 (5)	20 (15)	47 (35)	50 (38)	10 (8)
The lack of options for sending students to municipality-based services	134	4 (3)	20 (15)	18 (13)	61 (46)	31 (23)
The lack of coordinated services	132	4 (3)	12 (9)	39 (30)	48 (36)	29 (22)
The lack of funding	134	4 (3)	10 (7)	30 (22)	46 (34)	44 (33)
Mental health issues are not considered as a role of the school	133	14 (11)	32 (24)	29 (22)	39 (29)	19 (14)
Competing priorities	133	9 (7)	29 (22)	32 (24)	41 (31)	22 (17)
The belief that mental health problems do not exist	134	52 (39)	42 (31)	25 (19)	9 (7)	6 (4)

## Data Availability

Data generated during and/or analysed during the study are not publicly available due to privacy and ethical restrictions.
